# The Effect of Domiciliary Professional Oral Care on Root Caries Progression in Care-Dependent Older Adults: A Systematic Review

**DOI:** 10.3390/jcm12072748

**Published:** 2023-04-06

**Authors:** Elisabeth Morén, Pia Skott, Kristina Edman, Nivetha Gavriilidou, Inger Wårdh, Helena Domeij

**Affiliations:** 1 Department of Dental Medicine, Karolinska Institute, 141 04 Huddinge, Sweden; 2 Public Dental Service, Folktandvården Region Dalarna, 791 29 Falun, Sweden; 3 Centre for Clinical Research Dalarna, Uppsala University, 791 82 Falun, Sweden; 4 Public Dental Services, Folktandvården Stockholm AB, 118 27 Stockholm, Sweden; 5 Academic Centre for Geriatric Dentistry, 112 19 Stockholm, Sweden; 6 Administrative Centre for Public Dental Service, 791 29 Falun, Sweden; 7 Department of Surgical Sciences, Odontology & Maxillofacial Surgery, Uppsala University, 751 85 Uppsala, Sweden; 8 Department of Health Sciences, Karlstad University, 651 88 Karlstad, Sweden; 9 Health Technology Assessment—Odontology, Faculty of Odontology, Malmö University, 205 06 Malmö, Sweden

**Keywords:** dental personnel, domiciliary, root caries, fluoride

## Abstract

With care dependency, untreated root caries lesions (RCLs) and irregular dental visits are common. RCLs, if left untreated, could lead to pain, tooth loss, difficulties eating, and impact on general health. Therefore, there is a need for prevention and effective treatment for RCLs, and especially in those with care dependency. The aim of this systematic review was to investigate the effect of domiciliary professional oral care on root caries development and progression, in comparison with self-performed or nurse-assisted oral care. A literature search was conducted in four databases in November 2022. Two authors independently screened the literature throughout the review process. Five of the identified studies were found to be relevant. Four of these were assessed as having moderate risk of bias and were included in the review, while one study had high risk of bias and was excluded from further analyses. Due to heterogenicity of the included studies (and of the interventions and outcomes), no meta-analysis or synthesis without meta-analysis (SWiM) was performed. The participation of dental personnel performing mechanical plaque removal and fluoride, or chlorhexidine application seems beneficial for care-dependent older adults with risk of RCLs development and progression. However, future studies are needed.

## 1. Introduction

Among care-dependent older adults living in nursing homes, the number of untreated root caries lesions (RCLs) is high [1,2]. A systematic review by Zhang et al. [3] discovered several risk predictors for developing RCLs. The risk predictors stated were the age of 60 years or older, having poor oral hygiene, smoking, gingival recession, low socioeconomic status, and previous dental caries experience [3]. The risk for developing RCLs increases with age due to gingival recession caused by age and/or periodontal disease leading to exposed root surfaces [4]. RCLs are located on exposed roots of teeth and are of two categories. The first category is shallow and saucer-shaped and can be healed mechanically by toothbrushing with fluoride toothpaste [5,6,7]. The second category has the same shape as the first but is deeper and should be restored because of the risk of pulp exposure [7]. Bashir [8] reports a prevalence of untreated root caries among independent adults aged 20 years and older of 10.1%, with the highest prevalence found in the age groups of 70 years and older (12.5%) [8]. In other reports, prevalence of RCLs among adults, and older adults, varied broadly between 3.7% and 100% [3,9,10]. The daily oral hygiene procedure recommended by the Swedish dental guidelines is to brush the teeth twice per day with toothpaste containing 1450 ppm sodium fluoride [11]. According to the guidelines, the recommendations for prevention of RCLs are toothbrushing with high fluoride toothpaste containing 5000 ppm sodium fluoride, rinsing with 0.2% sodium fluoride solution, fluoride gel administered in mouth guards, and fluoride varnish or fluoride topical agents applied by dental personnel [11]. Interventions such as fluoride varnish applied every third month by dental personnel or self-brushing with high fluoride toothpaste have been shown to have a positive effect on arresting and preventing RCLs in care-dependent older adults living in nursing homes [12,13]. The definition of “care dependency” is “dependence on care in those who are young, ill, elderly or disabled, and dependent on another” [14].

Care-dependent individuals may therefore need help with basic care in daily living, for example with eating, personal hygiene including oral hygiene, transport outside or inside the home, and intake of medicine. Individual requirement of assistance with basic care is often related to the need for assistance with daily oral care [15].

Domiciliary dental care (DDC) is a service provided by dental personnel offering dental care at home, such as check-ups, preventive care, uncomplicated tooth extractions, simple/provisional tooth restorations, and adjustments of removable dentures [16,17]. Discontinued dental care attendance generally increases with age [18] and with cognitive impairment [19]. Further, with untreated RCLs the risk of pain, tooth loss, and eating difficulties could impact on general health [20]. Also, the risk of poor oral hygiene increases with age because of disease and disability [21]. With today’s high number of teeth with advanced prosthodontic reconstructions [21], performing nurse-assisted oral care for care facility/nursing home residents is challenging [22]. Therefore, there is a need for prevention and effective treatment of RCLs among older adults, and especially among those who are care-dependent [20,23]. Domiciliary dental care could therefore be beneficial for frail older adults wanting to stay at home. The effect of DDC, and especially the effect of dental professional oral care on root caries development, has, to our knowledge, not yet been explored in care dependent older adults.

The aim of the present systematic review was to investigate the effect of domiciliary professional oral care on root caries development and progression in care-dependent older adults, in comparison with self-performed or nurse-assisted oral care.

## 2. Materials and Methods

This systematic literature review was registered in the international prospective register of systematic reviews (PROSPERO). The protocol can be accessed at www.crd.york.ac.uk/PROSPERO/display_record.asp?ID=CRD42021274595 (accessed on 14 September 2021), see Supplementary Materials. All six authors (E.M., P.S., K.E., N.G., I.W., and H.D.) participated in the reviewing process.

### 2.1. Inclusion and Exclusion Criteria

#### 2.1.1. Inclusion Criteria

The review question was: What effect does domiciliary professional oral care have on root caries development and progression compared with oral care as usual in care-dependent older adults? The information was sorted into the categories population (P), intervention (I), comparison (C), and outcome (O) [24], as follows:

**P:** Individuals aged 60 years and older with at least one natural tooth. In addition, subjects must be care-dependent and not affected by congenital and/or acquired psychiatric disease.

**I:** Mechanical plaque removal, interproximal measures, as well as fluoride agents, and other preventive measures performed outside a dental clinic by dental personnel (dental nurse, dental hygienist, dentist).

**C:** Oral care as usual (self-performed or nurse-assisted).

**O:** Root caries development and progression, expressed as a caries index, for example the decayed, missing, and filled teeth (DMFT) index [25], the root caries index (RCI) [26,27] or the RCI described by Fejerskov et al. [28].

Only peer-reviewed, controlled clinical trials performed in humans (randomized controlled trials (RCTs), non-randomized controlled trials, and systematic reviews) written in Swedish, Norwegian, Danish, English, French, or German were included, with no limitations regarding the year of publication.

#### 2.1.2. Exclusion Criteria

Qualitative studies were not included in the review.

### 2.2. Search Strategy

The literature search was conducted by a librarian using four databases, PubMed, Cochrane Library, Cinahl, and MEDLINE via OVID. A search string was designed by a librarian with the support of the Library at Falun Hospital, Falun, Sweden.

The full search strategy can be found at: https://www.crd.york.ac.uk/PROSPEROFILES/274595_STRATEGY_20210913.pdf (accessed on 14 September 2021), see Supplementary Materials. A preliminary search was performed in June 2021 for testing and modifying the search string, followed by a discussion in the review group with the librarian. It was unanimously concluded that the search string was complete. Thereafter, the final searches were performed at the beginning of September 2021 and repeated in April 2022 and November 2022. Titles, abstracts, and full-text articles of the literature identified by the search strategy were screened independently by all the authors reading in pairs. The software Rayyan.ai [29] was used for the screening process. All studies of potential relevance according to the inclusion criteria were obtained in full text and the same author pairs independently assessed them for inclusion. Any disagreements were resolved by discussion.

### 2.3. Quality Assessment and Overall Risk of Bias

A Swedish version [30] of the Cochrane risk-of-bias (RoB) tool for non-randomized trials (ROBINS-I) and randomized trials, version 2 (RoB 2) [31] was used to assess the quality of the included studies. Using the RoB tools, the studies were classified as low, moderate, or high RoB. This part of the screening process was performed independently by all the reviewing authors reading in pairs and any disagreements were resolved through discussion.

### 2.4. Data Extraction and Data Analysis

Data extraction from the included studies was performed independently by two authors (E.M. and H.D.) and discussed in detail within the review group. Name(s) of the author(s), publication year, country, study design, participant characteristics such as age, gender, and number of teeth, number of participants, type of intervention, baseline measurements, and length of follow-up were extracted from each study. Due to heterogenicity of the studies included, in terms of both interventions and outcomes, no meta-analysis or synthesis without meta-analysis (SWiM) was performed.

## 3. Results

### 3.1. Search Results

**Figure 1:**
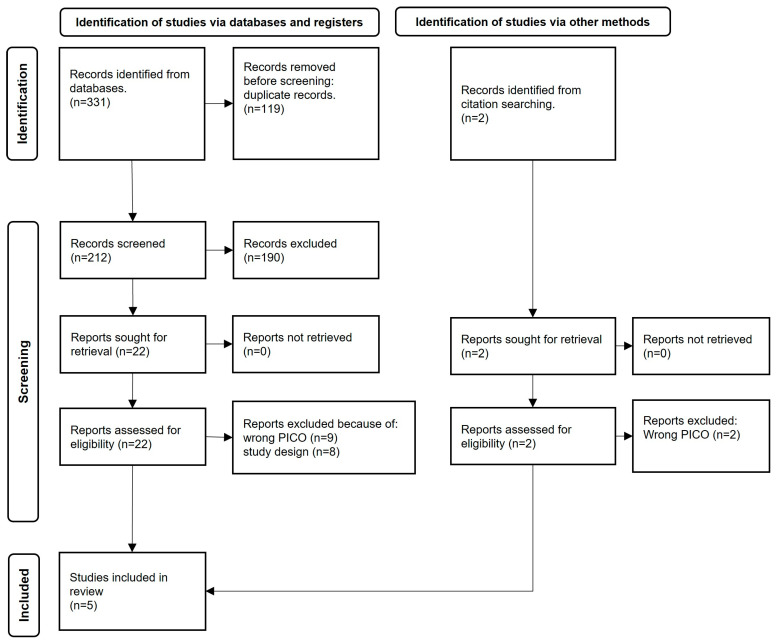
PRISMA flowchart of identification, screening, and inclusion of studies for the literature review [32].

Based on the designed search string, a total of 212 records were identified in the electronic databases. After screening of the titles and abstracts according to the inclusion and exclusion criteria, 190 records were excluded. The remaining 22 records were sought for retrieval and assessed for eligibility in full text. In addition, the reference lists of the included records were also screened. Two additional reports were identified through this process and included for full-text assessment. Out of these 24 records, five studies were found to be relevant (Figure 1) and were included for RoB assessment. The studies that were excluded are listed in Table 1. The main reason for exclusion was “wrong PICO”.

### 3.2. Risk of Bias

The five included RCT studies were assessed using the RoB 2 tool [31]. Four out of five studies were conclusively deemed to have moderate RoB; the fifth study had high RoB (Table 2). Only the results from the studies with a moderate RoB were further analyzed. The characteristics of these four included studies are summarized in Table 3.

### 3.3. Interventions

All four included studies [52,53,54,55] were RCTs studying the effect of various interventions for prevention and/or arrest of RCLs in care-dependent older adults. The study subjects were followed for different length study periods, from 3 months to 3 years. The interventions were: (1) professional tooth brushing every second week; (2) fluor protector varnish (Cervitec^®^) application at baseline and at 6, 13, 26, and 39 weeks; (3) professional cleaning with sodium fluoride varnish (Duraphat^®^), application, and oral hygiene instruction (OHI) once a month; and (4) OHI in combination with Cervitec or Duraphat application every 3 months, or application of silver diamine fluoride (SDF) every 12 months (Table 3).

### 3.4. Analysis

The four studies included in this systematic literature review all had different outcomes and different study population sizes, and further differed in the interventions administered, in data measurements, and study duration. Therefore, owing to the heterogenicity of the included studies, no meta-analysis or SWiM was performed. The findings for each study are therefore presented separately and in a narrative manner.

#### Root Caries Index

Barbe et al. [52] and Tan et al. [55] used the DMFT index and the five-level RCI, respectively. In addition, Tan et al. [55] also used the DFS root score. Girestam Croonquist et al. [54] used Fejerskov et al.’s five-level RCI [28], while Brailsford et al. [53] measured the length/distance from the gingival margin, the height, and width of the RCLs (Table 3).

### 3.5. Root Caries Development and Progression

Barbe et al. [52] found, in favor of the intervention group (professional brushing every second week by dental nurse), less development of RCLs at 3 months (*p* = 0.002). During the same period from baseline to 3 months, RCIs increased in the control group (*p* = 0.006) (Table 4).

Girestam Croonquist et al. [54] used Fejerskov et al.’s five-level RCI for grouping root caries scores, namely: score 1—healthy root surface and/or no RCLs; scores 2 and 4—inactive initial and manifest RCLs; scores 3 and 5—active initial and manifest RCLs. For no RCLs and initial RCLs, improvements were seen for both groups throughout the study period. Between 3 and 6 months, improvement of active RCLs was reported for the intervention group (*p* = 0.05) (Table 4).

Brailsford et al. [53] detected no new RCLs from baseline to 52 weeks in either group and concluded that RCLs in both groups were unchanged or had improved (Table 4). Contrarily, regarding height and width, the RCLs in the control group had increased compared with the intervention group (*p* < 0.001).

Tan et al. [55] followed their study subjects for 3 years. After 3 years, the group with only OHIs had a higher mean number of root surfaces with new active root caries or fillings compared with those with OHIs in combination with Cervitec or Duraphat or SDF application (Table 4). The relative risk for developing new RCLs was lower in the intervention groups compared with the controls. Furthermore, no difference in prevention of new RCLs was found between applying chlorhexidine (CHX) (four times per year), sodium fluoride (four times per year), and 38% SDF annually for a 3-year period. Compared with only OHIs, the application groups had a lower risk of developing new RCLs. Also, groups with higher risk of developing root caries were detected among the study subjects. The groups with higher risk of developing root caries were denture users (denture type not specified, probably partial dentures or dentures in only one jaw) (*p* = 0.021), study subjects with higher visible plaque index (VPI) score and higher root caries experience at baseline (*p* = 0.001), and exposed root surfaces (*p* = 0.001).

## 4. Discussion

This systematic review aimed to investigate the effect of domiciliary professional oral care interventions on root caries development and progression, in comparison with self-performed or nurse-assisted oral care.

The four included studies investigated different intervention methods over a variety of intervals and study periods. However, all the studies showed a positive effect in reducing or arresting RCLs [52,53,54,55]. The parameters shared by all the studied interventions were participation of dental personnel in mechanical plaque removal combined with application of fluoride agents (varnish or toothpaste) or CHX. This combination may be beneficial for care-dependent older adults with risk of RCLs development and progression. The effectiveness of an intervention with fluoride varnish and topical agents for reduction of RCLs depends on the interval and the frequency of application. For example, sodium fluoride varnish (Duraphat) is recommended to be applied four times per year [11,12]; however, a frequency of twice a year has also been shown to be effective in reducing the number of RCLs in care-dependent older adults [50]. Tan et al. [55] performed an intervention using SDF only once a year; even at this relatively low rate of application, they were able to report greater effectiveness in reducing new RCLs incidence compared with the other substances (Duraphat or Cervitec) [55].

The successful use of SDF among children for preventing coronal caries [23] has led to increased interest in studies among the older population with annual application of 38% SDF [20,23,55]. Despite promising results for arresting RCLs, SDF is only approved for use in some countries [56]. After application, SDF leaves a dark discoloration of the caries lesions [57].

### 4.1. Active Participation of Dental Personnel

Professional dental hygiene interventions have shown advantageous effects on oral hygiene status and management of RCLs within a relatively short time interval such as 2 weeks [52] or 1 month [54]. Regular and effective oral hygiene routines are important for care-dependent older adults. In addition, despite the costs of the intervention, a visit by a dental nurse could serve as a reminder for oral self-care. Hesitation to spend more for oral hygiene services was observed among the study subjects and their relatives in Barbe et al. [52], who report that participation would have been compromised if it had not been free of charge.

### 4.2. Training Nursing Staff

Education and hands-on training for both care-dependent older adults and nursing staff could contribute to improving oral hygiene among those in need [17]. Nurse-assisted oral care, particularly oral hygiene, is tedious and requires meticulous attention [16,49,58], particularly in view of the higher tendency of older people to retain their own teeth in high ages, and in view of complex dental constructions [21]. Moreover, nurse-assisted oral care for nursing home residents with poor cognitive function and communication problems is challenging [22]. Therefore, training in oral health care skills may be more useful than theoretical education for health care personnel [59,60]. To our knowledge, improved oral hygiene and compliance to perform such oral care routines over time has not been noted. One possible reason could be the high turnover rate among nursing staff in care of care-dependent older adults [61].

### 4.3. Awareness and Information among the Older Adults

There is a lack of prioritization of oral health, both among care-dependent older adults and among the nursing staff [62]. This could be attributed to insufficient knowledge of the connection between poor oral health and systemic diseases [2]. It is an important task for the dental profession to provide this knowledge before or in the early stages of care dependency. Failing to do so could lead to problems and high costs for health and dental care at both individual and societal levels [21].

Even though we had fairly open inclusion criteria, it was hard to identify studies that matched these. We found great diversity in study design, choice of intervention, and reported outcomes in the included four studies. When outcomes and outcome measurements differ, making it impossible to perform a meta-analysis, it may not be possible to improve future patient care. For future studies in this field, one possible way of using similar outcome measurements is, for example, to use the Core Outcome Measures in Effectiveness Trials (COMET) database to improve the efficiency of clinical trials and outcome measures. This database can be used to develop core outcome sets (COSs) [63]. COSs allow us to compare and collate results from various studies so that the basis for decisions, for both patients and dentalcare personnel, could be strengthened. Additionally, international consensus on appropriate interventions and outcome measurements would increase the feasibility of performing a meta-analysis of selected RCT studies involving clinical management of RCLs in frail older adults. A meta-analysis is crucial for closing the knowledge gap and conducting a clinical protocol for managing RCLs in frail older adults. 

## 5. Conclusions

Based on the available literature it is not possible to conclusively determine the effect of domiciliary professional oral care on root caries development and progression in care-dependent older adults. Future studies in this field should use a standardized protocol for RCT studies with similar study design, interventions, outcomes, and follow-up periods to enable comparison and meta-analysis.

## 6. Clinical Relevance

There is a need to investigate the current scientific knowledge of the effect of domiciliary professional oral care interventions to prevent and arrest progression of RCLs. A great proportion of care-dependent older adults are prone to this condition, which calls for identification of evidence-based strategies to address the issue among this population.

This review highlights the importance of dental personnel and optimal mechanical plaque removal with fluoride or CHX application in impeding or arresting RCLs. Adequate timely information and awareness about the risk of RCLs, prevention, and management methods are therefore crucial, ideally before the person enters the phase of care dependency.

## 7. Supporting Information

Contact was made with the author Anna-Grete Barbe regarding the number of participants included in her and her co-authors’ study. The right number was 50 (25 in each group). They recruited one more person after randomization.

## 8. Difference between Protocol and Review

Root caries development and progression on buccal root surface were initially the primary outcome measures. This was changed to root caries development and progression measured by a caries index.

## Figures and Tables

**Table 1:** Excluded reports sought for retrieval and with reason for exclusion.

Author, Year	Title	Where Published	Reason for Exclusion
Wyatt, 2009, [33]	A 5-year follow-up of older adults residing in long-term care facilities: utilization of a comprehensive dental program	Gerodontology. 2009;26(4):282–90.	Wrong PICO
Wyatt and MacEntee, 2004, [34]	Caries management for institutionalized elders using fluoride and chlorhexidine mouth rinses	Community Dentistry & Oral Epidemiology. 2004;32(5):322–8.	Wrong PICO
Niessen, 2012, [35]	Chlorhexidine varnish, sodium fluoride varnish, and silver diamine fluoride solution can prevent the development of new root caries in elders living in senior homes in Hong Kong	Journal of Evidence-Based Dental Practice. 2012;12(2):95–6.	Wrong study design
López, Uribe, Rodríguez, and Casasempere, 2013, [36]	Comparison between amine fluoride and chlorhexidine with institutionalized elders: a pilot study	Gerodontology. 2013;30(2):112–8.	Wrong PICO
Yi Mohammadi, Franks, and Hines, 2015, [37]	Effectiveness of professional oral health care intervention on the oral health of residents with dementia in residential aged care facilities: a systematic review protocol	JBI Database System Rev Implement Rep. 2015;13(10):110–22. https://doi.org/10.11124/jbisrir-2015-2330.	Wrong study design
ClinicalTrials.gov, 2018, [38]	Effectiveness on SDF solution and PVP-I combined NaF varnish in preventing root caries in elders	Available online: https://clinicaltrials.gov/ct2/show/NCT03654820 (accessed on 10 September 2021)	Wrong study design
Mojon, Rentsch, Budtz-Jørgensen, and Baehni, 1998, [39]	Effects of an oral health program on selected clinical parameters and salivary bacteria in a long-term care facility	Eur J Oral Sci. 1998;106(4):827–34.	Wrong PICO
Ritter, 2013, [40]	The efficacy of fluoride on root caries progression may be dose-dependent	Journal of Evidence-Based Dental Practice. 2013;13(4):177–9.	Wrong study design
Marchesan, Byrd, Moss, Preisser, Morelli, Zandona et al., 2020, [41]	Flossing is associated with improved oral health in older adults	Journal of Dental Research. 2020;99(9):1047–53.	Wrong PICO
Barbe, Küpeli, Hamacher, and Noack, 2020, [42]	Impact of regular professional toothbrushing on oral health, related quality of life, and nutritional and cognitive status in nursing home residents	International Journal of Dental Hygiene. 2020;18(3):238–50.	Wrong study design
MacEntee, Silver, Gibson, and Weiss, 1985, [43]	Oral health in a long-term care institution equipped with a dental service	Community Dentistry & Oral Epidemiology. 1985;13(5):260–3.	Wrong study design
Pearson and Chalmers, 2004, [44]	Oral hygiene care for adults with dementia in residential aged care facilities	JBI Library of Systematic Reviews. 2004;2(3):65–113.	Wrong PICO
Al-Nasser and Lamster, 2020, [45]	Prevention and management of periodontal diseases and dental caries in the older adults	Periodontology 2000. 2020;84(1):69–83.	Wrong study design
Patel, Khan, Pennington, Pitts, Robertson, Gallagher, 2021, [46]	Protocol for a randomized feasibility trial comparing fluoride interventions to prevent dental decay in older people in care homes (FInCH trial)	BMC Oral Health. 2021;21(1):1–12.	Wrong PICO
TrialSearch.com, 2017, [47]	A randomized controlled trial to evaluate the cost effectiveness of prescribing high concentration fluoride toothpaste to prevent tooth decay in older adults	Available online: https://www.who.int/trialsearch/Trial2.aspx?TrialID=ISRCTN11992428. 2017. (accessed on 10 September 2021)	Wrong PICO
Ekstrand, Poulsen, Hede, Twetman, Qvist, and Ellwood, 2013, [13]	A randomized clinical trial of the anti-caries efficacy of 5000 compared to 1450 ppm fluoridated toothpaste on root caries lesions in elderly disabled nursing home residents	Caries Research. 2013;47(5):391–8.	Wrong PICO
Raghoonandan, Cobban, and Compton, 2011, [48]	A scoping review of the use of fluoride varnish in elderly people living in long term care facilities	Canadian Journal of Dental Hygiene. 2011;45(4):217–22.	Wrong study design
Wikstrom, Kareem, Almstahl, Palmgren, Lingstrom, and Wardh, 2017, [49]	Effect of 12-month weekly professional oral hygiene care on the composition of the oral flora in dentate, dependent elderly residents: a prospective study	Gerodontology. 2017;34(2):240–8.	Wrong PICO
Jabir, McGrade, Quinn, McGarry, Nic Iomhair, Kelly et al., 2022, [50]	Evaluating the effectiveness of fluoride varnish in preventing caries amongst long-term care facility residents	Gerodontology. 2022;39:250–6.	Wrong PICO
Ekstrand, Martignon, and Holm-Pedersen, 2008, [51]	Development and evaluation of two root caries controlling programmes for home-based frail people older than 75 years	Gerodontology. 2008;25(2):67–75.	High risk of bias (RoB)

**Table 2:** Assessment of risk of bias (RoB), using the RoB tool, version 2 (RoB 2), of the five included studies, by domain and conclusive judgement.

RoB 2	Randomization process	Deviations from the intended interventions (effect of assignment to intervention)	Deviations from the intended interventions (effect of adhering to intervention)	Missing outcome data	Measurement of the outcome	Selection of the reported result	Conflict of interest	Conclusive judgment
**Ekstrand et al., 2008** [51]	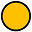	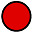	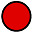	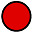	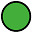	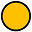	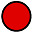	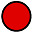
**Barbe et al., 2019** [52]	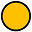	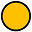	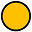	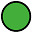	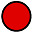	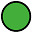	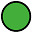	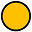
**Brailsford et al., 2002** [53]	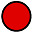	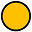	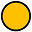	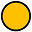	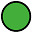	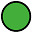	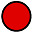	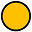
**Girestam Croonquist et al., 2020** [54]	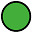	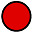	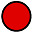	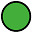	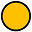	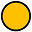	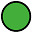	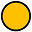
**Tan et al., 2010** [55]	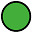	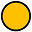	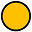	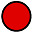	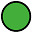	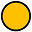	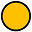	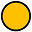

Green = low RoB; yellow = moderate RoB; red = high RoB.

**Table 3:** Characteristics of the included studies.

Author(s)	Barbe, Kottmann, Derman, and Noack [52]	Brailsford, Fiske, Gilbert, Clark, and Beighton [53]	Girestam Croonquist, Dalum, Skott, Sjögren, Wårdh, and Morén [54]	Tan, Lo, Dyson, Luo, and Corbet [55]
**Year**	2019	2002	2020	2010
**Country**	Germany	Great Britain	Sweden	China
**Title**	Efficacy of regular professional brushing by a dental nurse for 3 months in nursing home residents—a randomized, controlled clinical trial.	The effects of the combination of chlorhexidine/thymol- and fluoride-containing varnishes on the severity of RCLs in frail institutionalized elderly people.	Effects of domiciliary professional oral care for care-dependent elderly in nursing homes—oral hygiene, gingival bleeding, root caries and nursing staff’s oral health knowledge and attitudes.	A randomized trial on root caries prevention in elders.
**Aim**	To investigate the impact of professional brushing, performed every 2 weeks by a dental nurse, on the number of teeth, incidence of root caries, and further short-term oral health parameters, compared with residents whose oral hygiene was performed or supervised by staff according to standards of care corresponding to German law concerning the care for the elderly.	To determine the effect of a fluoride-containing varnish (Fluor protector) in combination with either Cervitec or a placebo varnish on the clinical characteristics of existing RCLs.	To describe the effects, for nursing home residents, of professional cleaning, and individual OHIs provided by registered dental hygienists, in comparison with daily oral care as usual.	To compare the effectiveness of the following four methods in preventing new root surface caries: (1) only OHIs every 3 months; (2) OHIs and applications of Cervitec varnish every 3 months; (3) OHIs and application of Duraphat every 3 months; (4) OHI and annual application of SDF solution.
**Study design**	RCT	Randomized double-blind longitudinal study	RCT	RCT
**Primary outcome**	Number of teeth	Root caries	Bleeding on probing (BoP), measured using the modified sulcus bleeding index (MSB)	Development of new caries on the exposed sound root surfaces of participants during the study period
**Number (*n*) of participants at baseline**	*n* = 50	*n* = 121	*n* = 146	*n* = 306
**Mean age, yrs, ± standard deviation**	83 ± 8	I = 85.6 ± 1.3 C = 79.8 ± 1.4	88.9 ± 4.1	78 ± 6.2
**Gender**	Female *n* = 34 Male *n* = 16	Female *n* = 65 Male *n* = 37	Female *n* = 108 Male *n* = 38	Female = 233 Male = 73
**Mean number of teeth**	17 ± 9	I = 13.73 ± 1.07 C = 15.50 ± 1.06	20.2 ± 3.0	14.3 ± 6.5
**Study duration**	3 mo	52 wks	6 mo	3 yrs
**Number (*n*) of dropouts**	*n* = 14	*n* = 19	*n* = 22	*n* = 103
**Root caries index (RCI)**	RCI (RCI1–RCI5), DMFT index	Length/distance from gingival margin, height, and width.	Fejerskov et al.’s five-level RCI [28]	RCI, DFS root score
**Time of data examination**	B + 3 mo	B + 13 wks + 26 wks + 1 yr	B + 3 mo + 6 mo	B + 1 yr + 2 yrs + 3 yrs
**Intervention, and number (*n*) of participants at baseline**	Professional brushing every second week by dental nurse, *n* = 25	Fluor protector varnish with Cervitec at baseline and at 6, 13, 26, and 39 wks, *n* = 52	Monthly professional cleaning, individual OHIs, and information, *n* = 72	(1) OHI + Cervitec every 3 mo, *n* = 71; (2) OHI + Duraphat every 3 mo, *n* = 80; (3) OHI + SDF every 12 mo, *n* = 72
**Intervention performed by**	Dental nurse	Dentist	Dental hygienist	Dentist
**Control and number (*n*) of participants at baseline**	Oral care as usual or nurse-assisted, *n* = 25	Fluor protector varnish with placebo at baseline and at 6, 13, 26 and 39 wks, *n* = 50	Oral care as usual or nurse-assisted, *n* = 74	OHI—placebo (water) every 12 mo, *n* = 83
**Risk of bias (RoB)**	Moderate	Moderate	Moderate	Moderate

B = baseline; C = control group; Cervitec^®^ = chlorhexidine (CHX) varnish; DFSs = decayed and filled surfaces; DMFT index = decayed, missing and filled teeth index; Duraphat^®^ = sodium fluoride varnish; I = intervention group; mo = month (s); *n* = number of study subjects; OHI (s) = (individualized) oral hygiene instruction (s); RCI = root caries index; RCL = root caries lesion; RCT = randomized controlled trial; SDF = silver diamine fluoride; wk(s) = week(s); yr(s) = year(s).

**Table 4:** Results regarding root caries lesions (RCLs) in the included studies, from baseline to 3 years’ follow-up.

Author(s), Year, Country	Results per Study							
**Barbe et al., ** **2019** [52], **Germany**	**Study groups**		**Baseline**		**3 mo**		**New RCLs**	
	I¹ group mean (SD) for RCI		1.1 (1.2)		1.3 (1.3)		RCI increased in the control group between baseline and 3 months (*p* = 0.006).	
	C group mean (SD) for RCI		1.5 (1.8)		2.6 (1.3)			
	*p*-value		0.433		0.002 *			
**Brailsford et al.,** **2002** [53], **Great Britain**	**Baseline—1 yr**							
	No new RCLs were detected in either the I^2^ or the c/placebo group.							
**Girestam Croonquist et al.,** **2020** [54], **Sweden**	**RCI**		**Baseline—3 mo**		**Baseline—6 mo**		**3–6 mo**	
			I^3^	C	I^3^	C	I^3^	C
	**Healthy** ***n* (%)**	Deteriorated	20 (28.6)	24 (39.3)	22 (31.9)	15 (27.3)	15 (21.7)	9 (16.4)
		Unchanged	38 (54.3)	27 (44.3)	32 (46.4)	26 (47.3)	33 (47.8)	28 (50.9)
		Improved	12 (17.1)	10 (16.4)	15 (21.7)	14 (25.5)	21 (30.4)	18 (32.7)
		*p*-value	0.41		0.84		0.76	
	**Initial caries** ***n* (%)**	Deteriorated	15 (21.4)	18 (29.5)	20 (29.0)	14 (25.5)	20 (29.0)	10 (18.2)
		Unchanged	44 (62.9)	30 (49.2)	39 (56.5)	31 (56.4)	38 (55.1)	36 (65.5)
		Improved	11 (15.7)	13 (21.3)	10 (14.5)	10 (18.2)	11 (15.9)	9 (16.4)
		*p*-value	0.29		0.82		0.39	
	**Active caries** ***n* (%)**	Deteriorated	9 (12.9)	3 (4.9)	7 (10.1)	9 (16.4)	11 (15.9)	11 (20.0)
		Unchanged	40 (57.1)	37 (60.7)	41 (59.4)	32 (58.2)	46 (66.6)	42 (76.4)
		Improved	21 (30.0)	21 (34.4)	21 (30.4)	14 (25.5)	12 (17.4)	2 (3.6)
		*p*-value	0.28		0.55		0.05 *	
**Tan et al.,** **2010** [55], **China**	**Study groups**	**Mean number/SE of new active root caries or fillings in each study group**						
		1 yr (*n* = 247)	2 yrs (*n* = 227)		3 yrs (*n* = 203)			
	OHI	1.5 (SE 0.2)	2.0 (SE 0.3)		2.5 (SE 0.5)			
	OHI + Cervitec	1.0 (SE 0.2)	1.0 (SE 0.3)		1.1 (SE 0.2)			
	OHI + Duraphat	0.8 (SE 0.2)	0.9 (SE 0.2)		0.9 (SE 0.3)			
	OHI + SDF	0.4 (SE 0.1)	0.7 (SE 0.2)		0.7 (SE 0.2)			
	All groups	0.9 (SE 0.1)	1.2 (SE 0.1)		1.3 (SE 0.2)			

C = control group; Cervitec^®^ = chlorhexidine (CHX) varnish every 3 months; Duraphat^®^ = sodium fluoride varnish every 3 months; I = intervention group; I¹ = professional brushing every second week by dental nurse; I^2^ = Fluor protector varnish with Cervitec at baseline, and at 6, 13, 26, and 39 weeks; I^3^ = monthly professional cleaning, individual oral hygiene instructions, and information; mo (s) = month (s); *n* = number of study subjects; OHI (s) = (individualized) oral hygiene instruction (s); RCI = root caries index; SD = standard deviation; SDF = silver diamine fluoride every 12 months; SE = standard error; yr(s) = year(s). * *p* ≤ 0.05.

## Data Availability

Not applicable.

## Supplementary Materials

Supporting information can be downloaded at: www.crd.york.ac.uk/PROSPERO/display_record.asp?ID=CRD42021274595 (accessed on 14 September 2021).
